# Lower Limb Wearable Capacitive Sensing and Its Applications to Recognizing Human Gaits

**DOI:** 10.3390/s131013334

**Published:** 2013-10-01

**Authors:** Enhao Zheng, Baojun Chen, Kunlin Wei, Qining Wang

**Affiliations:** 1 Intelligent Control Laboratory, College of Engineering, Peking University, Beijing 100871, China; E-Mails: zhengenhao@pku.edu.cn (E.Z.); chenbaojun@pku.edu.cn (B.C.); 2 Motion Control Laboratory, Department of Psychology, Peking University, Beijing 100871, China; E-Mail: wei.kunlin@pku.edu.cn

**Keywords:** wearable gait sensors, human body capacitance, capacitive sensing, muscle shape changes, human normal gaits, pattern recognition

## Abstract

In this paper, we present an approach to sense human body capacitance and apply it to recognize lower limb locomotion modes. The proposed wearable sensing system includes sensing bands, a signal processing circuit and a gait event detection module. Experiments on long-term working stability, adaptability to disturbance and locomotion mode recognition are carried out to validate the effectiveness of the proposed approach. Twelve able-bodied subjects are recruited, and eleven normal gait modes are investigated. With an event-dependent linear discriminant analysis classifier and feature selection procedure, four time-domain features are used for pattern recognition and satisfactory recognition accuracies (97.3% ± 0.5%, 97.0% ± 0.4%, 95.6% ± 0.9% and 97.0% ± 0.4% for four phases of one gait cycle respectively) are obtained. The accuracies are comparable with that from electromyography-based systems and inertial-based systems. The results validate the effectiveness of the proposed lower limb capacitive sensing approach in recognizing human normal gaits.

## 1. Introduction

Capacitive sensing is a useful technology for the measurement of multiple types of signals [1–4]. The capacitance of the simplest capacitor, which consists of two non-contact metal plates, depends on the size of the metal plates and the distance between them [2]. The relationship between capacitance and its relevant parameters can be used to measure distance, conductivity, pressure, *etc.*, with various applications to pressure sensors [5,6], artificial skin [7,8] and object detecting [9,10].

This characteristic has another important application: sensing human movements. A capacitance-based electrocardiograph system with a fixed sensor array has been proposed for heartbeat measuring [13–15], and promising results were obtained. In addition, the capacitance sensing concept has been used in human perspiration measurement [16] and respiration monitoring [17–19]. Furthermore, several studies have applied capacitive sensing in human activity recognition. Rekimoto proposed a capacitance-based approach for detecting human gestures, and the prototype was implemented with a wristwatch [20]. Two gestures were recognized by the proposed device. Cheng *et al.*, presented a concept for using capacitance sensing in human activity recognition and designed a prototype [21].

Lower limb locomotion mode analysis is important in human gait monitoring [22,23], injury prevention and evaluation [24,25], as well as control of powered lower limb prostheses and exoskeletons [26–28]. Previous studies on recognizing lower limb locomotion modes by wearable systems are realized mainly in two ways: using electromyography (EMG) sensors [29–31] and using inertial sensors [32–34]. However, there are several limitations for recognizing locomotion modes using the apparatus mentioned above. The EMG signals are so weak (with the amplitude at the microvolt-level) that they are difficult to be extracted from a noisy background [35,36]. As a consequence, in order to obtain valuable EMG signals, filtering, amplifying and other complex processing approaches are needed, which put forward a high expense of circuit design. For the inertial sensors, the signals can be full of noise (as the shifting of the sensors). In order to acquire higher accuracy, sensors with an independent source of noise have to be integrated on different spots of the human body to provide complimentary information [21].

Human muscle contractions not only result in changes of EMG signals and movements of limbs, but also lead to leg shape changes. The capacitance signals can reflect these changes of leg shapes during locomotive movements, and they carry characteristic information about gait mode states and gait phases. As a representation of movement intention, these signals lag behind neural signals recorded directly from cortex, but closely match EMG signals [4]. Based on this concept, we proposed a capacitive sensing-based method for locomotion mode recognition. Several confounding factors in classification (classifiers, analysis window length, leg dominance, *etc.*) were studied [4]. The results indicated that this method is a promising solution for locomotion mode classification. However, only limited motion modes that were important for lower limb amputees were investigated. No effort has been made on recognizing human daily gaits, for example, ramp ascent/descent and jogging. Moreover, the factors that are important for daily use and their impacts on capacitive sensing performance have not yet been studied.

In this paper, we present the specific design of the system for measuring human body capacitance and evaluate its effectiveness to recognize lower limb locomotion modes of human daily activities. The design of the proposed system is introduced in detail, which includes sensing bands of body capacitance, a signal processing circuit and a gait event detection module. Experiments on long-term working stability, adaptability to disturbance and locomotion mode recognition were carried out. With selected feature sets and an event-dependent linear discriminant analysis classifier, the proposed system can achieve comparable performance in recognizing human locomotion modes with current EMG-based systems and systems with inertial-based modules. The proposed approach offers a new solution for locomotion mode recognition of human daily gaits.

This paper is organized as follows. In Section 2, we describe the measurement of human body capacitance. Section 3 introduces the prototype system. Section 4 presents the application to recognizing lower limb locomotion modes. The system stability and performance of pattern recognition are validated by experiments described in Section 4. We conclude in Section 5.

## 2. Human Body Capacitance Measurement

**Figure 1.**
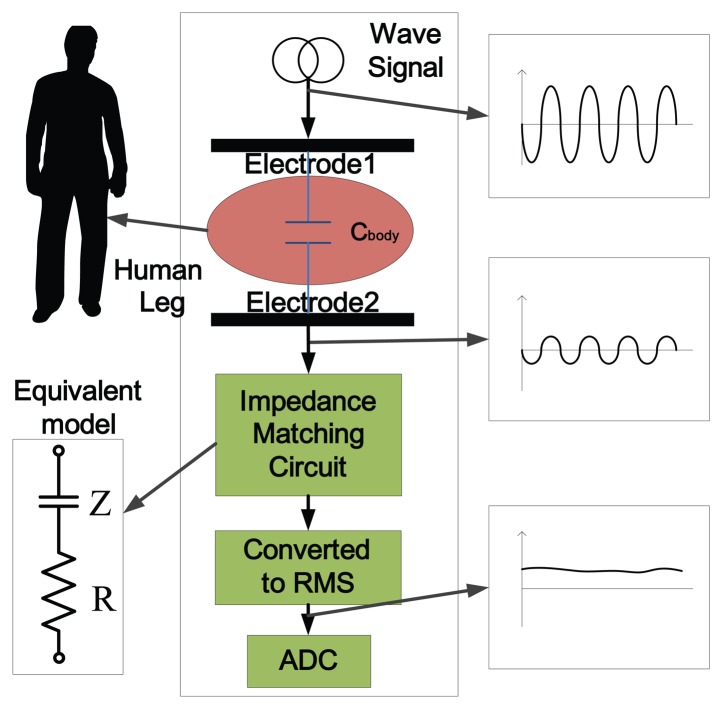
Design concept of the proposed capacitance-based sensing system for human locomotion recognition. The magnitude of the signal on the receiver electrode is influenced by the body movement. The signal was converted to root mean square (RMS) value before the analog-to-digital converter (ADC).

During human locomotion, the leg shape changes all the time, which is driven by stereotypical, but well-coordinated muscular contractions. In order to obtain the information of human locomotion by capacitive sensing, the first challenge is to record these shape changes of the thigh and the shank with capacitors. A capacitor is a device that can store energy in an electric field. The forms of capacitors vary widely depending on the specific use, but all of them contain at least two electrical conductors separated by a dielectric. In this study, as shown in Figure 1, the human body was considered as the dielectric, and two metal films were used as the electrodes. The capacitance is determined by a number of factors, such as the area of the electrodes, the distance and the dielectric constant between electrode pairs. When an electrode's area is constant or changes very little, the capacitance mainly depends on the material and the distance between the electrodes, *i.e.*, the shape, the structure and the material properties. Therefore, the muscle shape changes can be reflected by the capacitance signals.

### 2.1. Related Work

Before introducing the proposed method of capacitive sensing in this paper, we first present a brief review of current methods in capacitive sensing.

#### 2.1.1. Artificial Skin and Pressure Sensors

For artificial skin and pressure sensors, capacitive sensing technology was used to measure the distributed pressures [7,8] or shear forces [5]. In [7], each sensing element was built with a three-plate capacitive force sensor. The authors designed the sensing circuit with multivibrator chips and a row-column addressing technique for the sake of measuring as many sensor elements as possible, while in [8], the capacitance-to-digital chips were used to sample the capacitance signals. In [5], a sensor array was built. The authors separated the capacitive plates with an air gap. The flexible hemispheric structure made it possible to measure pressures and shear forces. A scanning circuitry was designed in this paper to record the signals.

#### 2.1.2. Object Detecting

Object detecting includes proximity detecting [9] and object tracking [10]. Both of the tasks can be achieved by detecting the changes of the electric field. In [9], capacitance sensing technology was used to detect the occupant. Both electrodes were fixed on a seat forming a capacitor. The capacitance can reflect the electric field changes that were caused by the occupant. The authors of [10] determined the positions of the object based on the information of the mutual capacitance of a few fixed electrodes. To sample the signals, the authors designed the capacitance-to-voltage converter based on trans-impedance amplifiers.

#### 2.1.3. Human Activity Detection

Many researchers focused on human activity detection [13–21], including human movement detection (walking, gesture, *etc.*) and physical condition monitoring (heartbeat, respiration, *etc.*). For physical condition monitoring, human skin was used as a capacitance electrode, because of the human body's conductivity. For the studies of human movement detection, the sensing devices should be fixed on the human body for stable measurement. Therefore, the capacitance arrays were used. To read the signals, the authors of [14] designed signal processing electronics containing an impedance converter, an amplifier and a filter circuit. While in [17] and [21], the authors used a colpitts oscillator to detect the frequency changes of the signals.

### 2.2. Measuring Body Capacitance

In this paper, the capacitance, *C_body_*, consists of two metal plates and the human body (see Figure 1). Electrode 1 is stimulated by a wave signal at a frequency of several hundred kilohertz, and peak-to-peak voltage remains constant. With a proper matching circuit, the equivalent impedance can be reflected by the magnitude of the signal extracted from Electrode 2. The impedance, *Z*, of the capacitor is a function of the capacitance:

(1) $$Z={\left(j\omega C_{\text{body}}\right)}^{-1}$$

where *ω* is the frequency of the driven signal, and it stays constant in the system. The resistor, *R*, in series and the capacitance, *C_body_*, make an impedance matching circuit. The voltage across the resistor will vary with *C_body_*. Thus, the signal of Electrode 2 can be extracted as the capacitance signal. The raw signal is a sinusoid wave with constant frequency (100 *kHz*) and varying amplitude, which is unsuited for direct sampling. Therefore, the signal is converted to root mean square (RMS) voltage before the analog-to-digital converter (ADC). We can acquire more information of human motion with an array of capacitors. The muscle deformation and contact conditions (tightness between the electrodes and skin) can be reflected by the change of capacitance. With proper regulation, the magnitude of the signals on the electrodes will vary with human movement. By measuring the voltage, we can get the current motion information.

## 3. Prototype

Based on the design concept mentioned above, the proposed capacitive sensing system is made up of four parts: two sensing bands, a signal processing circuit, a gait event detection module and a computer to receive data streams.

### 3.1. Sensing Bands

**Figure 2.**
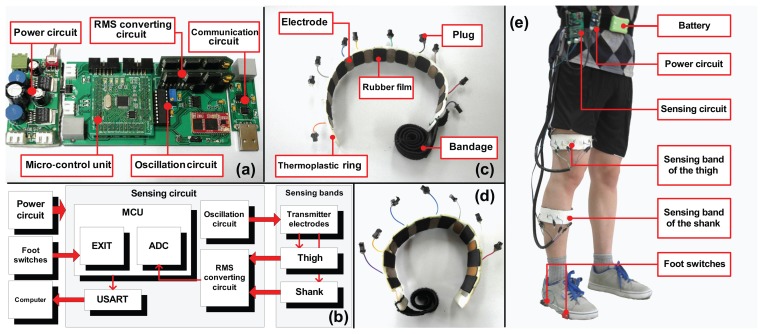
(**a**) shows the power circuit (**Left**), the sensing circuit (**Middle**) and the communication circuit (**Right**) designed for the system; (**b**) shows the architecture of the capacitive sensing system. The data flow is as follows: the sensing circuit first detects the capacitance signals and gait events and, then, transmits all the data to the computer; (**c**) and (**d**) show the sensing bands of the thigh (**c**) and the shank (**d**), respectively. The electrodes are made of copper pieces of 0.3 mm and fixed to the thermo-material ring by double-faced adhesive tape. The square of each copper film is about 5 cm × 2.5 cm. The black parts between the electrodes are rubber. The plugs connecting the electrodes are fixed on the outer surface of the rings, so that we can choose the key channels conveniently; (**e**) shows the placement of the system on human body. A Velcro tie with circuits and batteries is fastened to the waist. We used shielding lines to reduce the noise. Here, we only show the placement of the sensing bands on the right leg, which is similar to that of the left one.

In the proposed capacitive sensing system, as shown in Figure 2c, d, two separate arrays of capacitors were implemented on the sensing bands to record the shape changes of the thigh and the shank, respectively.

**Figure 3.**
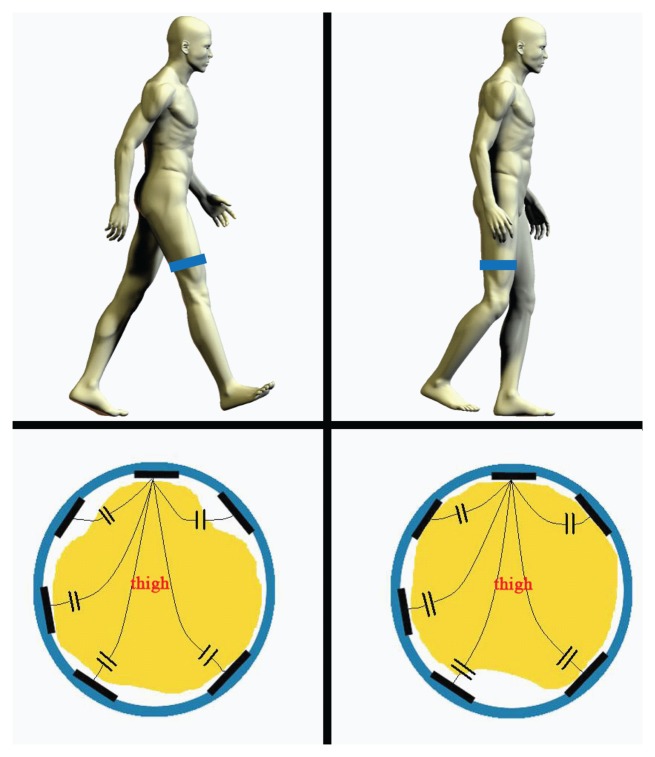
The sensing principle of lower limb movement detection based on our prototype. The top two figures show the flexion and extension of lower limb, and the bottom two figures show the cross-section of the thigh accordingly. The shape change of the thigh was reflected by the five channels of the capacitance signals. There are two capacitive sensing bands, which were placed on the thigh and shank, respectively. Here, we only show the sensing band on the thigh as an example.

Seventeen receiver electrodes made from copper sheets are mounted on the inner surface (seven for the shank and ten for the thigh) as the alternative electrodes. For each sensing band, there is one electrode connected to the signal source. All the other electrodes paired with this one, forming capacitors. The structure and sensing method of the capacitors are shown in Figure 3.

The muscular cross-section of the thigh changes visibly according to lower limb movement. The shape change of the lower limb can slightly change the relative positions between the electrodes and skin. This tiny change will have a strong effect on the capacitance signals.

In the implementation of sensing bands, the following factors should be taken into consideration. Firstly, the shape and distribution of muscles have an influence on capacitance signals. In addition, the skin condition and sweat also affect the signals of the sensing system. Moreover, undesired slipping degrades the performance of the system. To minimize the influence of these factors in this study, the sensing bands were made of thermoplastic material, which can be easily reshaped when it is heated up to about 70 °*C* and solidified when it is cooled to normal temperature. We reshaped the sensing bands in accordance with the shape of the lower limb to prevent the undesired shifting. In addition, we pasted insulating rubber at the intervals of electrodes to protect the electrodes from sweat. The sensing circuit can sample 10 channels of signals at most. We selected 10 channels that show large signal changes from all the alternative electrodes according to the subject (see below). Considering the individual difference in muscle distribution and leg width,

### 3.2. Circuits

The system architecture is shown in Figure 2a,b. The system consisted of the sensing circuit, the power circuit and the communication circuit. The sensing circuit was designed to process the capacitance signals from the sensing bands, including the oscillation module, the RMS-converting circuit and the control module. The circuit can process ten channels of signals at most. The oscillation module was built by using a waveform generator (MAX038) as its signal source and a high-slew-rate amplifier (TL3474) as the driver module. The waveform generator can perform a sinusoid signal at specific frequency with the peripheral circuit properly designed. The oscillation frequency is 100 *kHz*. To prevent waveform distortion, the signal was amplified by the driver module before applying the transmitter electrodes. The driver module also provided enough output current to the transmitter electrodes to be the signal source, although the current was several dozen milliamperes at most. We extracted the signals from the receiver electrodes and converted them to RMS voltage by an RMS-converting circuit. The regulated signals were then converted to digital data and processed by the control module. STM32 was used as the processor of the control module, which is an ARM-based 32-bit microprogrammed control unit (MCU) with a 10-channel 12-bit ADC imbedded. The MCU has low power consumption and high speed, which is well suited for applications requiring multichannel signal processing, as utilized in this study. The input voltage range of ADC is 0 *V* to 3.3 *V* . The power supply of the circuits is ±5 *V* which is converted from a 9.6 *V* Lithium battery by the power circuit. We also mounted foot switches on subjects' shoes to record foot contacts. A trigger signal was generated and sent to the sensing circuit whenever the foot switch contacts or leaves the ground. The communication circuit was designed for communication between the computer and the circuit.

As mentioned above, we measured the voltage across the body capacitors. The resistor divides the voltage with the body capacitor. To make the voltage vary in the range of 0 *V* to 3.3 *V* , the value of the resistors should be carefully adjusted. The excited frequency was 100 *kHz*, and the resolution of the ADC was 12-bit. With these parameters staying constant, the resistors determined the resolution and range of the capacitance. After a period of initial tests, we set the value of the resistor at 2.0 *k*Ω. The estimated capacitance range of each two electrodes is 0 *nF* to 30 *nF*. Then, the average dielectric constant measured in this study is about 60 to 80. The absolute capacitance does not affect the recognition performance. We just need to maintain the baseline as invariable during the experiment.

To guarantee the validity of the data, the flow of the sample data is conducted as shown in Figure 2b. We used the main circuit to process all the sample data, including capacitance signals and foot switch information. For the gait event detection board, the data was sent out only if there were changes on the switches. The main circuit updated and transmitted all the data of one sampling to the computer in two milliseconds. To reduce the error rate of communication, we utilized the Universal Asynchronous Receiver Transmitter (UART). The communication protocol is RS (Radio Sector)-232. Each data packet includes a data head and a sequence number. After each trial, the loss rate of data would be calculated based on the sequence numbers. If the loss rate was higher than 3 %, the data packet would be discarded, and the trial would be measured once more. The average loss rate was lower than 1 %. To reduce the random noise of the capacitance signals, a moving average filter (N = 5) was used.

### 3.3. Sensing Band Placement

To obtain more useful and reliable information about human motion, the following criteria of placing sensing bands on the human body are required:

(1) The sensing bands should be stable on the subject's leg during the experiments. Undesired slippage or shifting should be prevented.
(2) The placement of sensing bands on the human body should be comfortable without restraining human movement.
(3) Muscles around the receiver electrodes have distinct deformations, such that there are large signal changes.

Based on these rules, we placed the shank sensing band just above the muscle belly of the gastrocnemius, because it is easier to prevent the sensing band from sliding off and muscle deformation is obvious. For the thigh, we fixed the sensing band a few centimeters above the knee. In addition, to guarantee the stability of signal detection, we placed the electrodes that were connected with the signal source on bony areas or the spots with little muscle deformation. The sensing circuits and the batteries were fixed on subjects' waist to avoid restraining movement, which is shown in Figure 2e. Before placing the system on human body, the sensing bands were heated up to about 70 °*C* and surrounded the leg until solidification. The sensing bands were fastened on the leg with the bandage. There was Velcro on the bandage for manually adjusting the tightness. After the placement, the subjects were asked to familiarize themselves with the experiment. During this procedure, we manually selected five channels on each band that exhibited large signal changes for later measurements and analysis.

## 4. Human Normal Gait Recognition

Based on the capacitive sensing system described above, we can record the shape changes of the thigh and the shank with capacitors. In this section, we introduce the application of the capacitive system to recognize human daily gaits.

### 4.1. Classification Method

Capacitance signals, though time-varying, are quasi-cyclic. In other words, though capacitance signals change a lot for different gait phases of the same motion mode, the signals are similar at the same phase of a certain locomotion pattern. As a consequence, we used a event-dependent pattern recognition method, which is similar to the approach mentioned in [30]. Gait events of foot contact (FC) and foot off (FO) can be detected using two foot switches fixed on the shoe. One foot switch is placed at the position of the toe and the other one at the position of the heel. FC is determined when the state of at least one foot switch changes from “off” to “on”, while FO is determined when the states of both foot switches become “off” (the signal is “on” when the foot switch contacts the ground and otherwise, “off”). According to these two gait events, FC and FO, four phases were defined: prior to FC, after FC, prior to FO and after FO (they are marked as Pre-FC, Post-FC, Pre-FO and Post-FO, respectively). Different from the method that had been used in [30], we only used one analysis window for each phase in a gait cycle, rather than sliding analysis windows. For sitting, standing and squatting, there were no gait events, so we randomly selected analysis windows from the data of a trial. The window size was 200 ms for all the motion modes in this paper.

### 4.2. Feature Set and Classifier

In previous studies of motion mode recognition [30,31], linear discriminant analysis (LDA) was commonly used as the classifier for its good performance and low computational load. Time-domain features were often selected as the optimal features in recognition. Moreover, in our previous study [4], the LDA classifier and several time-domain features were chosen for the classification, and promising results were obtained on amputated subjects. In this paper, the LDA classifier was employed for locomotion mode recognition, and five time-domain (TD) features were chosen for candidate features, according to the following expressions: *f*_1_ = *avg*(*X*), *f*_2_ = max(*X*), *f*_3_ = min(*X*), *f*_4_ = *rms*(*X*), *f*_5_ = *std*(*X*), where *X* is the data matrix of the analysis window, *avg*(*X*) is the mean value of *X*, *std*(*X*) is the standard deviation of *X* and *rms*(*X*) is the root mean square of *X*, As a result, a 50-dimension feature value set was used for classifier training and testing.

### 4.3. Performance Evaluation

To make a more reliable evaluation of recognition performance, leave-one-out-cross validation (LOOCV) was used for the calculation of recognition error. Since ten measurement units were conducted in the experiment, the data in one unit were used as testing data and the data in the other nine units were applied as training data. This procedure was repeated ten times, and data in every unit were used once as the testing data.

The overall recognition error (*RE*) is calculated by:

(2) $$RE=\frac{N_{\text{mis}}}{N_{\text{total}}}\times 100\%$$

where *N_mis_* is the number of misrecognized testing data and *N_total_* is the total number of testing data.

To better illustrate the recognition performance of certain locomotion patterns, a confusion matrix was defined as:

(3) $$C=\left(\begin{array}{cccc} c_{1,1} & c_{1,2} & \ldots & c_{1,11}\\ c_{2,1} & c_{2,2} & \ldots & c_{2,11}\\ \ldots & \ldots & \ldots & \ldots \\ c_{11,1} & c_{11,2} & \ldots & c_{11,11} \end{array}\right)$$

where each element is defined as:

(4) $$c_{ij}=\frac{n_{ij}}{{\bar{n}}_{i•}}\times 100\%.$$

*n_ij_* is the number of testing data in mode *i* recognized as mode *j* and *n̄_i_*_•_ is the total number of testing data in mode *i*. A higher value of *c_ij_* (*i* ≠ *j*) denotes that it is easier for mode *i* to be misclassified as mode *j*.

## 5. Experimental Results

### 5.1. Subjects and Experiment Protocol

To evaluate the effectiveness of the proposed capacitive sensing system, we carried out two kinds of experiments. Firstly, we tested the system's long-term working stability, adaptability to disturbance and the performance of different sensing positions. One able-bodied subject was recruited in these experiments. The subject was 24 years old, 1.80 *m* in height and 74 *kg* in weight.

The first experiment was to test the system's stability in long-term measurement. The repeatability of the capacitance signals in different gait cycles of the same locomotion mode is a key factor that influences the recognition performance. After wearing the sensing bands for a while, sweat and slippage may occur, due to human movement. To test whether these factors cause large signal changes, a six-hour experiment was carried out. Three kinds of locomotion modes: sitting, standing and normal walking were monitored for the testing of the repeatability. The experiment was divided into 25 groups with a 15-minute interval, and in each group, there were five measurements. At the intervals of the experiment, the subject was free to undertake daily activities. To record the slippage of the sensing bands, we placed markers on the leg along the lower edge of the sensing bands. The subject's skin was dry at the beginning of the experiment.

The second experiment was to test the robustness of the sensing system. An accidental crash or hit during the experiment or daily use bring disturbance to the signals. To evaluate the system's adaptivity to disturbance, the subject was asked to sit on a chair in relaxation condition. The experimenter hit the rings from different directions to simulate disturbance in daily use. Six hits were carried out.

The third experiment was to evaluate the performance of the system in different sensing positions. The sensing band on the shank was placed at the position of the gastrocnemius. Since the muscle belly of the lower leg can prevent the sensing band from sliding down, the position was almost the same during each trial. However, the sensing band cannot be easily placed at the same point on the thigh in different trials. We performed a third experiment to evaluate the performance of the system while the sensing band was placed on different spots on the leg. One able-bodied subject was recruited in this experiment. The experiment was divided into two sessions with the sensing band placed on two different spots, and there was 5 cm of distance between them. During each session, six locomotion modes, standing, sitting, ascending stairs, descending stairs, climbing obstacles and normal walking, were performed alternatively. Each mode were performed five times. The gait cycle of all the motion modes was the same. In this experiment, the subject was required to finish all the six modes in one group instead of repeated testing of one motion mode.

We then carried out the second kind of experiment to evaluate the performance of the system in human gait recognition. Twelve able-bodied subjects participated in this experiment. They had an average age of 24.4 (±3.4) years, an average height of 1.76 (±0.05) m and an average weight of 69.3 (±10.8) kg. The subjects were randomly separated into two groups to wear the sensing bands either on their right leg or on their left leg, respectively. Eleven motion modes that could be frequently encountered in daily life were monitored in our experiment: standing (St), sitting (Si), squatting (Sq), normal walking (NW), ascending stairs (SA), descending stairs (SD), climbing obstacles (Ob), ascending a ramp (RA), descending a ramp (RD), side walking (SW) and jogging in place (Jog). For the tasks of standing, sitting and squatting, subjects were asked to stand still, sit on a chair of 42 cm in height or squat in their comfortable posture for 10 s, respectively. Ascending stairs and descending stairs were tested on a four-step staircase. The stairs were 75 *cm* in width, 40 *cm* in depth and 15 *cm* in height. Ascending ramp and descending ramp were measured on a slope with a length of 210 *cm* and a tilt angle of 30°. Obstacles were 40 *cm* in width, 18 *cm* in depth and 25 *cm* in height. The distance between two adjacent obstacles was 70 *cm*. The tested leg was required to pass over obstacles first, followed by the non-tested leg. Subjects were encouraged to walk at their favorite speeds for normal walking. For side walking, the subjects were asked to walk sideways in the direction of the tested leg. For the task of jogging in place, the subjects jogged for several steps at the same site. All the subjects participating in the experiments provided written and informed consent.

To evaluate effects of the resolution of the capacitance on recognition performance, we carried out another experiment. A subject was employed in this experiment. The experiment was divided in three sections. In each section, the subject was asked to perform the tasks in the same way as the above recognition experiment. We set the serial resistor to 1 *k*Ω, 2 *k*Ω and 4.7 *k*Ω for the three sections, respectively. A larger resistor value means higher resolution, but a smaller sampling range. The subject was allowed to take rests between the sections.

### 5.2. Long-Term Working Stability

**Figure 4.**
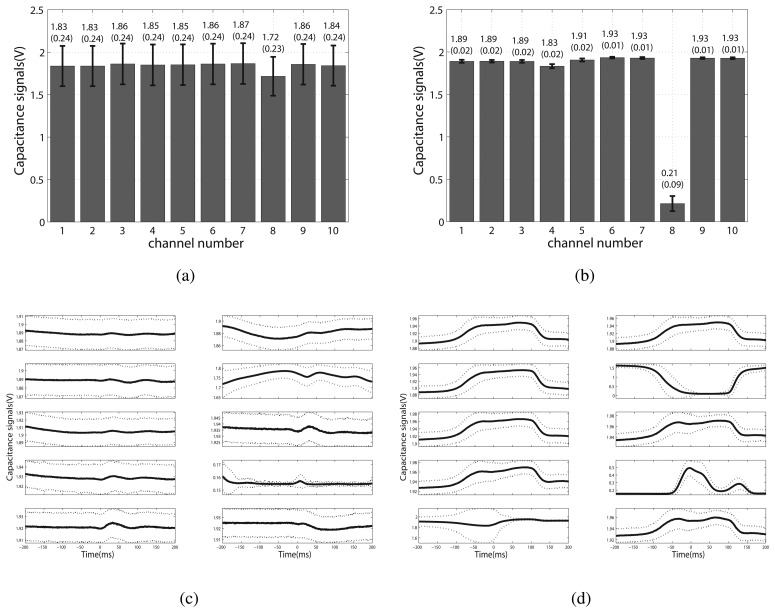
Continuous working stability of the capacitive system. (**a**) and (**b**) show the error bar of the capacitance signals of sitting and standing, respectively. The numbers on each bar represent the average voltage and its standard deviation; (**c**) and (**d**) demonstrate the average signals (solid lines) of four phases and the variation (dotted lines) over different gait cycles of normal walking. The zero point on the x-axis indicates the gait event, FC and FO, respectively. The data shown in (c) was Pre-FC and Post-FC, with a duration of 200 ms in each phase, while in (d), the data was Pre-FO and Post-FO.

With an experiment of six hours, 125 groups of data for each motion mode were acquired. The results are shown in Figure 4. Figure 4a,b demonstrate the average value and the variance of each channel of sitting and standing, respectively. Figure 4c,d show the average and variation of signals in four phases (Pre-FC, Post-FC, Pre-FO, Post-FO) during normal walking. Results can be concluded as follows:

(1) For the tasks of sitting and standing, the standard deviation values of the signals were small for most of the channels. The maximum variations of the two motion modes fell on channel eight (0.09 for sitting and 0.23 for standing, respectively). The classification of different locomotion modes was to calculate hyperplanes in a high-dimensional feature vector space. For channel eight, the large difference between sitting and standing was beneficial for classification. We also calculated the standard deviation for each trial of sitting and standing. The average values are 0.0035 V and 0.0019 V for sitting and standing, respectively. Although the current system has no complex shielding method, the results of noise analysis show that the current shielding method is acceptable.
(2) For the task of normal walking, the capacitance signals showed a similar tendency in different gait cycles. In this paper, the recognition method was based on gait phases. Therefore, we compared the capacitance signals extracted from the same phases of all the gait cycles (Figure 4). The tendency of the signal curve was clear, with a small standard deviation (0.1 *V* ) for most of the channels.
(3) For all the three tested motion modes, there were exceptional channels showing large variations (channel eight for sitting and standing and channel nine for normal walking, respectively). For sitting and standing, the distinction of the average value of channel eight was more prominent than the other channels. The muscle contractions cause changes in tightness between the electrodes and skin. Channel eight was more sensitive to motions, due to the electrode position on the thigh.
(4) There was no slippage for the sensing band of the shank. While there was slight slippage on the thigh, the maximum distance was about five millimeters, which was found on the back of the thigh. At the end of the experiment, sweat was observed on the thigh and shank. In order to test whether the slippage and sweat caused a signal change, we analyzed the average values of sitting and standing. One way repeated measure ANOVA was employed to compare the data of the first three groups and the last three groups. The difference was not statistically significant with, *p* = 0.125 for sitting and *p* = 0.583 for standing. The results showed that sweat and slight slippage do not affect the signals.

### 5.3. Adaptability to Disturbance

There was a step instantly after the disturbance for each channel. The signals then quickly recovered to normal levels in 200 *ms* to 500 *ms*. The change rate of the signals (*CR*) was defined as:

(5) $$CR=|\frac{{\text{ave}}_{2}-{\text{ave}}_{1}}{{\text{ave}}_{1}}\times 100\%|$$

where *ave*_2_ is the average value of one second after the disturbance, and *ave*_1_ is the average value of one second before the hit.

The average change rate was 0.58%, which is the level of noise. The maximum change rates were 13.80% and 11.35%, which occurred at the fourth and second trial on channel four. However, the average change rates change to 0.82% and 0.20% for the next post-disturbance second. Therefore, after the disturbance, signals can turn back to the normal level within a period of time.

### 5.4. Sensing Positions

In this experiment, the five time-domain features were fed to the LDA classifier. We used a phase-dependent method to segment the data of a gait cycle. Each phase size was set at 200 ms. Five-fold LOOCV was employed to evaluate the recognition results. We compared the average recognition accuracies of two measuring positions. There was a tiny difference between the two positions. The recognition accuracy of position 1 was 97.58%. For position 2, the recognition accuracy was 97.18%. The difference was less than 1%. The results show that the change of position within a limit does not make a large difference in the recognition accuracy.

### 5.5. Recognition Performance

To evaluate the recognition performance of the sensing system, 10-fold LOOCV was used. To acquire enough data for training and testing, the experiment was designed as follow. For the tasks of normal walking, side walking, jogging on the spot and obstacle climbing, there were ten trials with six gait cycles for each trial. While for the tasks of ascending/descending stairs and ascending/descending ramp, there were twenty trials with four gait cycles in each trial. The duration of sitting, standing and squatting was 10 s for each trial, and these modes were measured for ten trials. For the dynamic modes like normal walking and side walking, the first and the last gait cycles were discarded (While for ascending/descending stairs, only the first gait cycles were discarded.) At last, the data of 40 gait cycles for each motion modes was acquired.

For the tasks of ascending stairs/ramp, descending stairs/ramp, climbing obstacles, normal walking, side walking and jogging on the spot the segmentation of analysis windows was carried out using the event detecting method mentioned above. For the tasks of sitting, standing and squatting in which foot switch signals remained constant during experiments, analysis windows were evenly distributed along the time for each trial. The signals of these motion modes are nearly unchanged. Thus, this simplification for analysis window selection will not affect feature extraction as well as recognition performance significantly.

**Figure 5.**
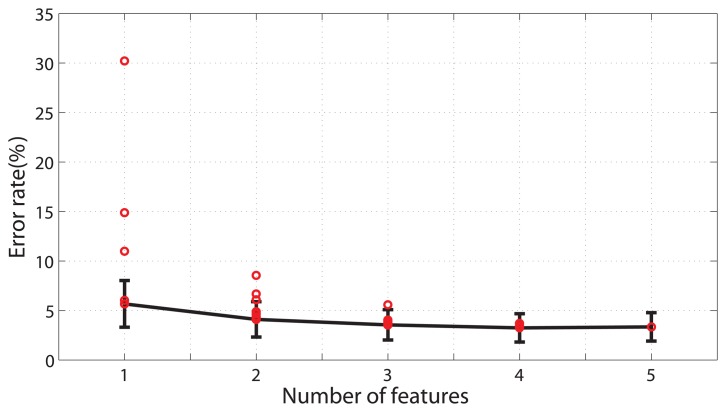
Classification errors of different combinations and numbers of features. The red dots represent the average classification errors of all the subjects in a specific number of features. The lowest error rate among the combinations is denoted as the black line. The error bar is the standard deviations of the subjects. The results are obtained by the LDA classifier with a 200-ms window size. These data were obtained from 12 able-bodied subjects.

Feature set was important in motion mode classifications, so the selection of features was carried out. As mentioned above, five basic time-domain features were chosen as the candidates to form the feature set. All the combinations of the features(31 combinations in total) were calculated and the results were shown in Figure 5. For the single features, *avg*(*X*) and *rms*(*X*) performed much better than the others with the average error rate being 6.0% and 5.7% respectively. While *std*(*X*) performed the worst with the error rate of 30.2%. The optimized combinations of features of all the numbers were compared with one-way repeated-measure ANOVA. The *p* values of pair-wise comparisons from one to five were *p* = 0.00, 0.001, 0.012, 0.238. The difference was not statistically significant between four features and the whole features. Taking the recognition accuracy and computational load into consideration, the optimized feature set was chosen as *avg*(*X*) + *max*(*X*) + *rms*(*X*) + *std*(*X*).

**Figure 6.**
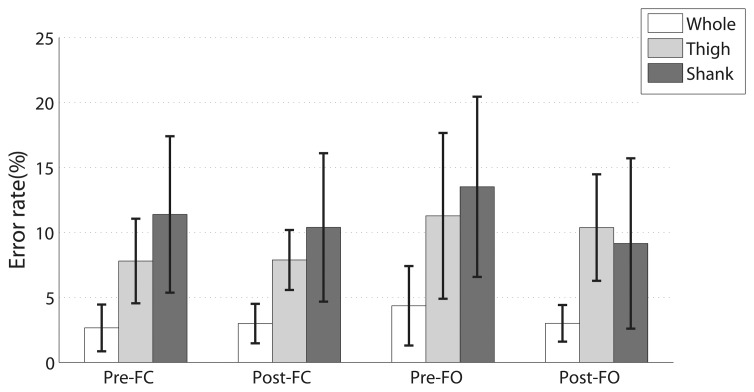
Classification errors of different combinations of signal channels. The results are obtained by LDA classifier with 200 ms window size. Data are obtained from twelve able-bodied subjects.

We compared the classification performance with signals from the thigh, the shank and both (Figure 6). For all the four events, classification using signals from both the shank and the thigh achieved the best performance. The differences were statistically significant for all the four events using one-way repeated-measure ANOVA. The performance of the thigh was significantly better than that of the shank in Pre-FC, while in other phases, the differences were not statistically significant.

Overall recognition accuracies for phase Pre-FC, Post-FC, Pre-FO, Post-FO were 97.3% ± 0.5%, 97.0%±0.4%, 95.6%±0.9% and 97.0%±0.4%, respectively. The detailed recognition results expressed by confusion matrices are shown in Table 2. Very low recognition accuracy was not observed in any motion mode. The lowest performance was observed in Pre-FO for recognizing normal walking from other motion modes, but the recognition accuracy is still as high as 88.6%. In addition, we compared the recognition performance between the measurements from left leg and right leg. The average classification accuracies were 96.7% ± 0.5% and 97.3% ± 0.3% for left leg and right leg respectively. The difference was not statistically significant (*p* = 0.855, one-way ANOVA). The measured leg dose not affect the recognition performance.

**Figure 7.**
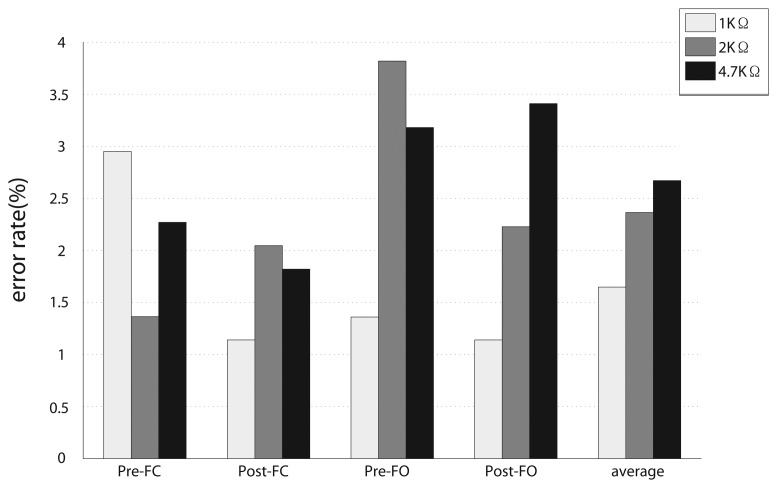
Classification errors of different levels of resolution. The right bars show the average recognition errors over four gait phases. The results were obtained with linear discriminant analysis (LDA) classifier and 200 ms phase size.

We then compared the average recognition accuracies in different resolutions. The classification method and parameters used were the same as those mentioned above (10-fold LOOCV, 200 ms phase size, four time-domain features and LDA classifier). The average recognition accuracies for the three levels of resolutions were 98.4% (1 *k*Ω), 97.7% (2 *k*Ω) and 97.4% (4.7 *k*Ω). The results (Figure 7) show that higher resolution may cause a slight drop in average recognition accuracy, but the influence was limited (with only a 1% difference). For four gait phases, there was no regular change with increased resolution. It can be concluded that adjusting the resolution within a range will not make a big difference to recognition performance, as long as the signal changes can reflect human motions well.

### 5.6. Discussion

This paper presents a wearable capacitance sensing method to recognize human normal gaits. After a series of experiments, we validated the stability of the system for long-term use, the stability with respect to disturbance and the performance in different sensing positions. Compared with EMG sensors and IMU (Inertial Measurement Unit) sensors, the sensing system achieved excellent recognition performance in recognizing human normal gaits. We proposed the system as an alternative to other kinds of sensors in applications related to recognizing human normal gaits.

The sensing method in our prototype was different from that in electrocardiograph (ECG) sensing [13–15], respiration monitoring [17,18] and pressure measuring [5,7]. A capacitor in ECG sensing consists of an electrode, an insulator and the skin [13]. By using this method, the system can perform well under the conditions of the human body being still and proper contact between the skin and the insulator. In [18], respiration is measured with a sensor belt on the chest. The volume change of the lungs during respiration changes the overlap area of the electrodes. In pressure measuring, the absolute value of the capacitance is recorded to calculate the absolute pressure. The sensing methods in the above applications cannot be directly transplanted to human movement detection for which the sensing front-ends were placed on human body. In addition to sampling as much motion information as possible, the negative impact caused by human motion should be minimized. Considering these restrictions, our prototype is built with two sensing bands, which can be reshaped based on leg shape. Only small slippage was observed after six-hours' use in our study, which demonstrated the effectiveness of the design.

**Figure 8.**
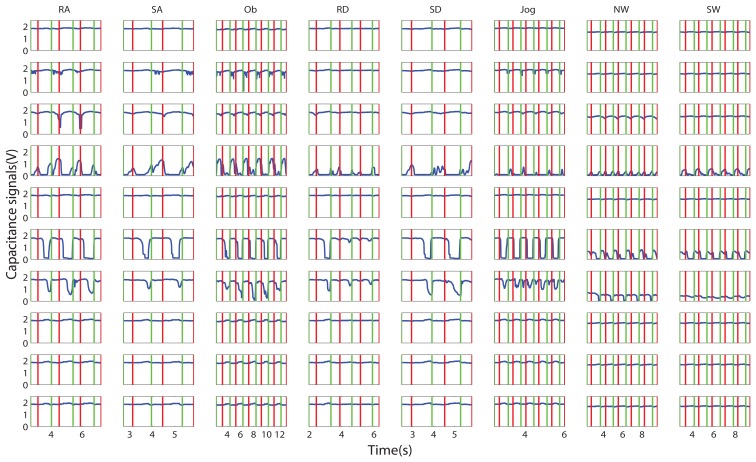
Capacitive signals of different locomotion modes. The x-axis is the time in seconds. The y-axis stands for the amplitude of the signals. The raw signals are shown as blue lines, while the red and green vertical lines stand for the gait events, foot contact (FC) and foot off (FO), respectively. Each row of the subfigures stands for one of the ten channels of the capacitance signals. Each column stands for a specific locomotion mode. The data for sitting, standing and squatting are steady lines with different amplitude, which are not shown in this figure.

In our study, impedance matching circuits were designed to read out the capacitance signals (Figure 1). The wave signals sampled from Electrode 2 were converted to RMS voltage before ADC. There was no amplifying or filtering procedure needed. While the sensing technique used in [17,21] was based on a colpitts oscillator, which detects the change of oscillation frequency, the reference frequency was 17 *MHz*, and the frequency change was several hundreds Hz [21]. To obtain enough motion information from such a high frequency, amplifying, filtering and a high resolution ADC (24-bit) were needed, which increased the system's cost. In our study, the signal processing module was simpler to implement, At the same time, the signals during locomotion were clearly observed, as well (see Figure 8).

The factors that may influence the system performance were analyzed based on the experiments. The long-term stability experiment evaluated the influence of slippage and sweat on the signals. Although rubber was pasted on the inner surface of the capacitance ring, several-millimeter drops of the sensing band on the thigh were still observed after six hours of experimentation. However, the signals were stable with small standard deviations. We compared the difference between the beginning part and the ending part for sitting and standing. The difference was not statistically significant. The experiment on adaptivity to disturbance demonstrated that the signals can recover to a normal level after hitting. Moreover, the recognition performance was not so sensitive to sensing positions as EMG sensors. The limitation of the current system is that the sensing bands were dressed directly on the leg. The obtrusiveness of dressing may hinder application in daily life. In further improvement of this hardware, this deficiency will be considered.

Our previous work [4] indicated that locomotion mode recognition based on the capacitance sensing method is an alternative tool for prosthesis control. It focused on the optimization of the parameters in motion mode classification, including analysis window length, classifiers, phase size, *etc.* The investigated motion modes are six kinds of gait modes, which are important for lower limb amputees. In this paper, side walking, squatting, ramp ascending, ramp descending and jogging in place were newly taken into consideration. These motion modes are frequently encountered in daily life and have physical significance in other related applications, for example, human activity monitoring. In this paper, the specific design of the sensing system was optimized in circuit and sensing bands, which make the system more stable for daily use than the former prototype. Factors that may occur in daily situations were analyzed to test the stability of the proposed system. Through able-bodied subject experimentation, we provided evidence that the capacitance signals are stable with long-term use and can recover within a second after disturbance. Moreover, the results of different sensing positions proved that the proposed system can achieve satisfactory recognition performance, with the position changing within a range. With only four features and a simple classifier, the recognition performance was comparable with that of EMG sensors and IMU sensors.

The capacitance sensing system is still promising for applications related to recognizing human normal gaits, like gait monitoring and exoskeleton control. Firstly, due to the recognition process used in this system, current gait modes can be obtained within a gait cycle. In this research, both the classifier and the feature set used for recognition are easy to implement and have low computational load. The recognition performance demonstrated that the system can offer excellent accuracy as benchmarked to EMG signals [30] and IMU sensors [32] for the same motion modes. Secondly, the signals of the system during long-term measurement were stable and can return to normal levels in less than 500 *ms* after disturbance. The recognition accuracy was not influenced by the change of the sensing positions in a limit. The results show that the system is promising for daily use. Thirdly, the system can recognize eleven normal gait modes that are frequently encountered in daily life, with only two sensing bands being placed on a unilateral leg. Apart from these, the sensing system is cheap, and the recognition performance is encouraging. Further research will be done to enhance the practicability of the capacitive sensing system.

In the present paper, four time domain features and an LDA classifier were used for off-line classification, which are both computationally efficient. With the phase-dependent method, four motion mode decisions can be made within a gait cycle. Future implementation in a wireless communication and embedded classification algorithm will enable real-time classification in daily life. Since, currently, we used only one set of sensing bands for all subjects, a reshaping procedure was needed before each experiment. For practical use in daily application, reshaping is only needed for first time usage. Then, the customization of the band can free the user from the reshaping procedure. In the future, we will try some other materials to replace the current thermoplastic one for practical usage. Moreover, the hardware can be optimized (wireless communication and customized sensing bands), so as to make it more compact and portable.

## 6. Conclusion

In this paper, we presented a capacitive sensing system for recognizing human normal gaits. The sensing system was simple in design and implementation, meanwhile providing satisfactory performance compared with EMG sensors and IMU sensors. The system measures leg shape changes during the human motion. Our experiments validated that the system is sufficient for recognizing human normal gait modes. As an alternative to EMG sensors and IMU sensors, the capacitance sensing method can be further improved through hardware design and more thorough experiments.

## Figures and Tables

**Table 1.** Change rate of the perturbation.

**Trial Number**	**Average Change Rate (%)**	**Maximum Change Rate (%)**	**Channel**
1	0.30	2.32	4
2	1.34	11.35	4
3	0.07	0.53	4
4	1.63	13.80	4
5	0.05	0.13	4
6	0.09	0.67	8

**Table 2.** Confusion matrix (mean (standard error of the mean)) for twelve subjects (%). RA, ascending a ramp; SA, ascending stairs; Ob, climbing obstacles; RD, descending a ramp; SD, descending stairs; Jog, jogging in place; NW, normal walking; SW, side walking; Si, sitting; Sq, squatting; St, standing.

		Estimation										

Phase	Target	RA	SA	Ob	RD	SD	Jog	NW	SW	Si	Sq	St

Pre-FC	RA	94.8(1.4)	0.2	0.0	1.7	1.3	1.3	0.2	0.4	0.0	0.0	0.2
	SA	0.0	97.1(1.4)	0.4	0.2	0.2	0.4	0.6	0.6	0.2	0.2	0.0
	Ob	0.2	0.0	98.2(0.6)	1.0	0.2	0.4	0.0	0.0	0.0	0.0	0.0
	RD	0.6	0.0	0.0	99.0(0.6)	0.2	0.2	0.0	0.0	0.0	0.0	0.0
	SD	1.0	0.0	0.0	0.4	94.6(1.9)	1.0	2.9	0.0	0.0	0.0	0.0
	Jog	1.0	0.0	0.0	0.2	0.6	95.5(1.3)	0.4	2.3	0.0	0.0	0.0
	NW	0.0	0.0	0.0	0.0	2.9	0.2	95.8(1.7)	1.0	0.0	0.0	0.0
	SW	0.2	0.0	0.2	0.0	0.0	1.3	1.0	97.3(0.8)	0.0	0.0	0.0
	Si	0.0	0.0	0.0	0.0	0.0	0.0	0.0	0.0	100.0(0.0)	0.0	0.0
	Sq	0.0	0.0	0.0	0.1	0.1	0.0	0.0	0.0	0.0	99.7(0.2)	0.0
	St	0.0	0.0	0.0	0.0	0.0	0.8	0.0	0.3	0.0	0.0	98.9(0.9)

Post-FC	RA	93.8(1.8)	0.6	0.4	0.4	1.7	1.9	0.0	0.4	0.6	0.0	0.2
	SA	0.0	97.5(1.1)	0.4	0.6	0.0	0.4	0.4	0.5	0.0	0.0	0.2
	Ob	0.4	0.0	98.3(0.6)	0.9	0.0	0.0	0.0	0.4	0.0	0.0	0.0
	RD	0.0	0.0	1.0	97.3(1.4)	0.0	1.5	0.0	0.0	0.0	0.0	0.2
	SD	0.2	0.0	0.4	0.2	93.5(2.3)	1.3	3.8	0.6	0.0	0.0	0.0
	Jog	2.3	0.2	0.0	0.6	0.0	95.3(1.5)	0.2	0.9	0.0	0.0	0.4
	NW	0.0	0.0	0.3	0.0	5.4	0.0	94.2(1.5)	0.1	0.0	0.0	0.0
	SW	0.6	0.2	0.0	0.2	0.0	1.5	0.0	97.5(0.6)	0.0	0.0	0.0
	Si	0.0	0.0	0.0	0.0	0.0	0.0	0.0	0.0	100.0(0.0)	0.0	0.0
	Sq	0.0	0.0	0.0	0.0	0.0	0.0	0.0	0.0	0.1	99.9(0.1)	0.0
	St	0.0	0.0	0.0	0.0	0.0	0.8	0.0	0.0	0.0	0.0	100.0(0.0)

Pre-FO	RA	91.1(1.9)	0.2	0.4	3.8	2.9	0.8	0.6	0.0	0.2	0.0	0.0
	SA	0.4	96.9(0.9)	0.0	0.7	0.2	0.0	0.2	1.3	0.0	0.2	0.1
	Ob	0.2	0.0	97.1(1.0)	1.2	1.0	0.0	0.2	0.0	0.0	0.0	0.2
	RD	2.1	0.2	0.2	96.9(1.1)	0.0	0.2	0.0	0.2	0.2	0.0	0.0
	SD	1.5	0.0	1.0	0.2	95.2(1.9)	0.2	1.9	0.0	0.0	0.0	0.0
	Jog	0.2	0.0	0.2	0.4	0.2	96.9(1.0)	0.2	1.7	0.0	0.2	0.0
	NW	1.5	0.2	1.7	0.4	5.2	0.0	88.6(3.2)	1.4	0.2	0.0	0.8
	SW	0.4	0.5	1.7	0.8	0.2	4.5	1.0	90.4(2.6)	0.0	0.4	0.0
	Si	0.0	0.0	0.0	0.0	0.0	0.0	0.0	0.0	100.0	0.0	0.0
	Sq	0.0	0.0	0.0	0.0	0.0	0.0	0.1	0.0	0.0	99.9(0.1)	0.0
	St	0.0	0.0	0.0	0.0	0.0	0.0	0.1	0.7	0.0	0.0	99.2(0.8)

Post-FO	RA	97.1(1.0)	0.6	0.0	0.6	0.2	0.6	0.0	0.2	0.6	0.0	0.0
	SA	0.7	97.3(1.3)	0.2	0.0	0.0	0.2	0.1	0.2	0.4	0.8	0.0
	Ob	0.0	0.0	96.3(1.4)	1.6	0.2	1.0	0.8	0.0	0.0	0.0	0.0
	RD	0.4	0.2	1.0	96.7(1.2)	0.2	0.2	0.8	0.2	0.0	0.0	0.2
	SD	1.0	0.2	0.0	0.2	96.0(1.1)	0.6	1.5	0.4	0.0	0.0	0.0
	Jog	0.8	0.0	0.0	0.0	1.3	95.9(0.9)	0.4	1.4	0.2	0.0	0.0
	NW	0.0	0.4	0.0	0.2	3.1	0.0	95.0(1.5)	0.8	0.0	0.2	0.2
	SW	0.0	0.0	0.4	0.0	0.0	4.2	2.0	93.4(2.3)	0.0	0.0	0.0
	Si	0.0	0.0	0.0	0.0	0.0	0.0	0.0	0.0	100.0(0.0)	0.0	0.0
	Sq	0.0	0.0	0.0	0.0	0.0	0.0	0.0	0.0	0.0	100.0(0.0)	0.0
	St	0.0	0.0	0.0	0.0	0.0	0.0	0.0	0.8	0.0	0.0	99.2(0.8)
